# The Hospital. Nursing Section

**Published:** 1906-10-20

**Authors:** 


### The Hospital.

### Nursing Section.

Contributions for "The Hospital," should be addressed to the Editor, "The Hospital" Nursing Section, 28 & 29 Southampton Street, Strand, London, W.C.

No. 1,048.—Vol. XLI. SATURDAY, OCTOBER 20, 1906.

### Notes on News from the Nursing World.

### THE NURSES' HOME AT MILLBANK HOSPITAL.

The new Nurses' Home at Millbank Military Hospital is still quietly progressing, though there is not the slightest chance that it will be ready for occupation until the late spring or early summer of next year. The alterations found necessary when Queen Alexandra realised the diminutive size of the rooms apportioned to the sisters has, of course, much retarded the work, but matters are getting sufficiently far advanced for onlookers to see for themselves the extra space placed at the disposal of the staff by the new arrangement. According to the old plan, the Home included twelve bedrooms for the servants, with a large servants' hall and big kitchens. A separate wing has now been erected by the side of the original building which will contain all these rooms, so that the defects to which we called attention last year will no longer exist. Fortunately there were no brick walls to take down between the rooms to enlarge them, as they are divided by solid partitions which are put up *en bloc*, and these are, it is stated, both fireproof and noiseproof.

### AFFILIATION TO THE JUBILEE INSTITUTE.

The members of the Shropshire County Nursing Federation have under consideration a proposal for affiliation to the Jubilee Institute. Miss Amy Hughes, superintendent of the institute, has been to Shrewsbury and has given an address to a large and influential gathering, including many members of the local associations which constitute the Federation. It appears to have been extremely well received, and some of the speakers, who had previously been doubtful as to the wisdom of joining the Institute, confessed that their objections had been removed. There was a good deal of discussion as to the advantages to be gained by the Federation, but with regard to the Institute Sir Offley Wakeman observed that he thought that the only aim of the authorities was to carry out the wishes of the late Queen to do all in their power to further nursing among the poor classes. This is an accurate interpretation of the position, and we are sure that if the Shropshire Federation, after due deliberation, decide upon affiliation they will find their hands strengthened.

### ALL SAINTS' SISTERS AND INDIAN TRAINED NURSES.

We insert with pleasure this week a letter from a correspondent who was trained at St. George's Hospital, Bombay, under the Sisters of All Saints', and is now a matron of a hospital, protesting against the reflections which she considers were cast in some "Notes from India" in our issue of September 15, on the work done by these devoted women. But we do not think that the writer of the article intended any slight, and we are sure that she would be the first to admit that nothing but praise is due to them for their unselfish pioneer labour. As "Trained in Bombay" says, the All Saints' Sisters had to use the material at hand when they fell short of better, and the Eurasian nurses on the staff, who are no longer employed, rendered useful service. It is also, we freely admit, quite possible, as our correspondent contends, for an Indian trained nurse to do the work required in the wards of English hospitals.

### AN INCIDENT WITH A MORAL.

There is a moral in the incident in a nurse's life related in our columns this week which, though it should be plain enough, is worth emphasising. The vivid story of the narrow escape of the author from death by burning points to the urgent necessity of every nurse on night duty being provided with a safety lamp. But this is not all. The nurse, whose life was so nearly sacrificed because the candle in her hand set light to some inflammable material which had been placed beneath the patient, had been placed alone at the end of the corridor in charge of a serious case her first night on duty. This was a great mistake. It may be desirable for a nurse new to night duty to learn from observation as soon as possible how to look after a patient in a dangerous condition. But no one who is not thoroughly experienced should be left alone through the night in charge of a serious case.

### THE NURSES' HOSTEL BOARD AND MISS HULME.

It is with much regret that we learn that the meeting of the directors of the Nurses' Hostel Company, Limited, on Friday week, has not had the result hoped for by many who anticipated that it would be followed by a settlement agreeable to all parties. In order not to interfere with that end, we held over last week a report of the meeting which we had received as well as several letters from correspondents interested. A few of these we now publish, with others which show how strongly the feeling prevails that Miss Hulme has not had fair play accorded to her. With regard to the proceedings at the abortive meeting, it need only now be said that when one of the deputation of nurses who were allowed to be present touched on the grave injury which she alleged had been done to Miss Hulme, she was told that that was not a question for the deputation to discuss, and that it was no more their business "than if the cook had been dismissed." However, the members of the deputation, before they were summarily dismissed without practically any discussion whatever of the purpose for which they had expressed a wish to attend, contrived to repudiate the charge against Miss Hulme that she had managed the hostel as a nurses' institution, and to affirm that the exact opposite was the case. They were also able to urge that as Miss Hulme is a nurse, if she suffered, they also suffered, and were bound to protest against the treatment accorded to her. The deputation, though greatly disappointed at their reception, did not abandon the expectation that a satisfactory communication would subsequently be forwarded to Miss Hulme from the directors, until it became clear that there was no intention of offering her the explanation which she claims. In fact, a most unsatisfactory letter has since been received from the Board by Miss Hulme's solicitor. There is, happily, yet a chance that the shareholders will insist upon justice being done. The annual meeting is to take place on Thursday, the 18th, at four o'clock, and though the existence of Miss Hulme is ignored in the circular convening the meeting, it is understood that the appointment by the Board of a new superintendent and secretary will be announced on the occasion. We hear that Miss Chamberlain, the secretary for a few weeks, is to be the new superintendent, and Miss Kett the new secretary. According to the annual report, there has been a decrease in the visits paid by nurses in the south block, which "is chiefly accounted for by the fact that some of the tenants' rooms are now occupied by masseuses who are in permanent residence." The truth is, we are informed, that there are only four masseuses in the south block, all the others being either general trained or maternity nurses. One of the causes for complaint has been that nurses who wished to sublet their rooms when they were away, were obliged to give up doing so, as the records were kept so incorrectly, and they could get no redress. This and other matters for complaint may well be brought before the meeting of shareholders, but a resolute, and, we trust, successful effort will at any rate be made to compel the Board to state explicitly why Miss Hulme was dismissed. As matters stand at present an apology is clearly due to her for the extraordinary course pursued.

### THE ADA LEWIS NURSES' INSTITUTE TO BE CLOSED.

We regret to announce the death of Mrs. Lewis-Hill which occurred on Saturday last at her residence, 16 Grosvenor Square, and also to learn that the Ada Lewis Nurses' Institute, which was founded by the deceased lady, will now be closed. Mrs. Lewis-Hill has left notable bequests to numerous charitable institutions, but no provision is made in her will for the Ada Lewis Nurses' Institute. It appears that her well-meant effort to help the sick poor of the middle classes and to relieve the congestion of the hospitals for the benefit of the general practitioner, proved a great disappointment to her. Several trained nurses, as our readers know, have been kept at the Institute and sent out for several hours a day to needy patients. The enterprise, however, contrary to the expectations of the founder, only obtained, it is stated, scant support from the medical profession, and Mrs. Lewis-Hill accordingly is said to have determined not to spend any more money upon the undertaking.

### A DISTRICT NURSE FOR EVERY PARISH IN SCOTLAND.

At the Conference of the Scottish District Unions which are affiliated with the National Union of Women Workers, a demand was put forward by Dr. Anne Mercer Watson, of Aberdeen, for a Midwives Bill for Scotland, which she said is, at any rate, required in certain districts. That there is room for improvement in midwifery, as practised in Scotland, can, no doubt, easily be shown, but the Scottish people will hardly, we think, begin to agitate for legislation on the lines of the Midwives Act now in operation on this side of the border, unless or until they are satisfied that it meets the necessities of the case. A suggestion more likely to commend itself to the community was made by the President, Lady Aberdeen, who urged the representatives of the Unions to do their utmost to help in "the establishment of a district nurse in every parish in Scotland." The high appreciation of the services of the district nurses already at work in Scotland, and the generous support given to existing nursing organisations, justifies the hope that Lady Aberdeen's ideal may be attained in the not very distant future.

### MASSAGE EXAMINATIONS FOR ORDERLIES.

It has been stated that "a written examination of nursing orderlies was held last week at the Royal Victoria Hospital, Netley, and an examination on anatomy and practical work would take place on Monday, October 15, in London, at the Military Hospital, Millbank." In order to prevent misconception we may say that a certain number of men are trained every year at Netley as masseurs for the Royal Army Medical Corps, and examined by the Society of Trained Masseurs prior to the certificate of the Society being granted. The examination of orderlies just held at Netley had reference only to the written portion of the Society's examination; the oral and practical part took place at the Millbank Military Hospital, London, on Monday. It is only during the last year or two that the massage which a soldier so often needs to bring a wounded limb or a damaged hand into active service once more has been given by the orderlies. Before then a civilian masseur had to be employed at considerable extra cost to the authorities. Now there is a continuous series of classes at Netley. When a new one is about to be formed the matron of each military hospital is asked to send in the name of any member of her orderly staff whom she considers likely to profit by training as a masseur. The selected candidates are then instructed at Netley for six months by a certificated member of the Incorporated Society of Trained Masseurs. The class is never larger than six, as it is impossible to give the special and separate instruction required to a greater number; on the last occasion there were only four candidates. The men who are successful in gaining their certificate either return to the staff from which they originally came, or are sent to another hospital where there is a vacant post for a masseur to be filled.

### THE COST OF COOKERY CLASSES.

The London County Council have notified to the authorities of the Poplar and Stepney Sick Asylum that they are unable to arrange to give the usual course of eighteen lessons in sick cookery to the nurses which the latter have formerly received, and must diminish the number to twelve or raise it to twenty-four. The acting matron of the Sick Asylum states, however, that twelve are insufficient, and twenty-four inconvenient and extravagant. She therefore suggested that an ex-County Council teacher, who instructs the nurses at Croydon Infirmary, and whose terms are the same as those of the Council, be asked to give a course of eighteen lessons at Poplar. The suggestion has been adopted, and Miss Baker, the teacher in question, is to receive a fee of 8s. 6d. per lesson and third-class travelling expenses. It cannot be said that the conditions of payment err on the side of extravagance.

### QUEEN ALEXANDRA'S MILITARY NURSING SERVICE.

We are officially informed that Sister E. A. Cox, of Queen Alexandra's Imperial Military Nursing Service, has been transferred to ss. *Plassy* for Indian Troopship Service, from the Queen Alexandra Military Hospital, Millbank; Sister M. L. Harris to the ss. *Plassy*, from the Military Hospital, Chatham; Sister M. Pedler to the ss. *Plassy*, from the Cambridge Hospital, Aldershot; Sister L. E. Mackay to Egypt, from the Military Hospital, Gosport; Sister E. M. Denne to South Africa, from the Royal Herbert Hospital, Woolwich; and Sister M. Walker to South Africa, from the Connaught Hospital, Aldershot. Staff Nurse G. S. Jacob has been transferred to the ss. *Plassy* for Indian Troopship Service, from the Connaught Hospital, Aldershot; and Staff Nurse W. L. Everingham has been posted to the Cambridge Hospital, Aldershot, on her appointment.

### NURSING CONFERENCE IN LONDON.

A conference is being organised by the Provisional Committee of the National Council of Nurses, to be held at the hall of St. George's, Hanover Square, in Mount Street, on November 22nd, 23rd, and 24th. We understand that the subjects to be discussed on each successive day are Tuberculosis, Maternity, and Mental Nursing, and that experts are being asked to read papers and join in the discussions. The chairman on the second day will be Dr. Champneys, President of the Central Midwives Board; and on the third, Dr. Robert Jones, President of the Medico-Psychological Association. The subjects will be illustrated by exhibits round the hall, to which admission will be free. Any nurse wishing to exhibit inventions on the occasion should communicate with Miss Eleanor C. Barton, matron, Chelsea Infirmary, Cale Street, S.W.

### THE PENSION FUND AND BRIGHTON NURSES.

An address on the subject of the Royal National Pension Fund for Nurses was delivered by the Secretary, Mr. Louis H. M. Dick, on Monday evening last week to a meeting of nurses at the Sussex County Hospital, Brighton. The board-room was courteously lent for the occasion, but proved inadequate, so large was the attendance. Amongst those present were Miss Scott, matron, Miss Myles, superintendent of the Union Infirmary; Miss Buckle, lady superintendent of the local branch of the Jubilee Institute; and, to the delight of her many friends, Miss Monk, late matron of King's College Hospital.

### NURSING AT BISHOP AUCKLAND.

We understand that Miss E. G. Wood, who from the outset was attached until recently to the Bishop Auckland Working Men's Nursing Association, is now engaged as a nurse on her own account in the town where she has been doing excellent work for more than twelve years. It was since she ceased to be connected with the Association that the committee found it advisable to amalgamate with the District Nursing Association as announced in our issue of October 6. Miss Wood has already obtained so much support that she has found it necessary to engage a colleague.

### KING EDWARD'S FUND FOR NURSES IN IRELAND.

At a meeting of the Council of King Edward's Fund for Nurses in Ireland, held last week in Dublin, the Hon. Secretaries reported that since the last meeting in April donations amounting to £2, and members' subscriptions to £3 10s. had been received. Applications for membership from six nurses were considered and accepted. The Council granted the sum of £10 to a nurse member to help her over convalescence after an operation.

### A TRADESMAN'S LOAN TO A NURSE-MATRON.

A very sad story was told at the Chesterfield County Police Court last week, when the late nurse-matron of the Marsden Moor Hospital was charged with obtaining the sum of £4 from a local fishmonger under false pretences. The guilt of the accused was admitted by her solicitor, who stated that she had been assisting some one whose name she would not reveal, with the result that she had been tempted to apply to the prosecutor to lend her money "to get things for the hospital," which, however, she had put into her own pocket. There was no excuse for her misconduct, though we are glad that the Bench, instead of sending her to prison, ordered her to refund the money, and to be bound over under the First Offenders Act. But we are surprised that the fishmonger complied with her request. He might reasonably have supplied goods to the institution on credit to her order. When he was asked to forward money to carry on the hospital he should have referred the matron to her employers.

### The Nursing Outlook.

"From magnanimity, all fears above;
From nobler recompense, above applause,
Which owes to man's short outlook all its charm."

### NURSING HOMES.

The opening of the new theatre and extension of the Acland Nursing Home at Oxford by Professor Osler on the 13th instant calls attention to the need which is felt in every town of any size for a well found nursing home or pay hospital. It is remarkable that the colleges at Oxford University have not provided a sanatorium or pay hospital for the use of the undergraduates, many of whom require such accommodation in the course of each term. In the United States the universities have shown much more regard for their undergraduates by the provision of suitable sanatoria. The Acland Home with its extensions will now supply the undergraduates with adequate accommodation. It is surprising that the colleges have not shown a more liberal spirit in making contributions to the building fund which still shows a deficiency of some £1,200, a fact which we commend to the earnest attention of these authorities. The new additions are well up to date in all respects, and the Acland Home may now be considered reasonably complete, whilst the garden and grounds, with the open balconies, offer advantages which are not always obtainable at nursing homes in cities. The message of congratulation and interest which Queen Alexandra sent to Miss Acland marks once more the gracious remembrance of old friends which Her Majesty invariably displays, and it naturally caused great enthusiasm at Oxford on Saturday.

Having succeeded financially the Home is now threatened with a crisis in its history by a request for the admission of patients who can pay something, though not a remunerative rate. There is a demand for accommodation for patients belonging to the lower middle classes who require hospital accommodation when they have to undergo surgical operations. The practitioners of Oxford are promoting a memorial to the committee of the Home, asking them to take patients in at a lower rate than four guineas per week, the present charge. We hope that the committee will have the wisdom to refuse to admit any one to the Home at less than a remunerative rate, though the charges should be made inclusive and be free from extras. It is perfectly true, no doubt, that the class immediately above the poor who are able to pay something, though not necessarily a remunerative rate, for the surgical aid they need, should be provided with adequate accommodation, and should have the privilege of being attended by their own doctors. Such patients when able to pay two or even three guineas a week are not eligible to occupy free beds in a voluntary hospital, and it is not just to the medical profession that they should lose the fees which such patients can afford to pay a private practitioner when ill. Still, any self-supporting nursing home, with a limited number of beds, must speedily experience financial disaster, if it endeavours to supply the needs of the lower middle classes who cannot pay a remunerative rate.

We have often pointed out that the best solution of the difficulty would be for each voluntary hospital to provide a pay wing to which such cases can be admitted, and where they can be attended, when necessary, by their own practitioners. The doctors of Oxford should, indeed, memorialise the Radcliffe Infirmary Committee, not that of the Acland Nursing Home. It should not be impossible or even very difficult for the Committee of the Oxford Voluntary Hospital to set apart one pavilion for paying patients, and this development of its work would mark a new departure for good, which could not fail to increase the popularity and financial strength of the Radcliffe Infirmary. In any case, the committee of the Acland Home will be wise to confine its work to the class of cases which has made that home a success in the past. This policy is especially desirable now that this Home has become, in fact, the sanatorium of the University, seeing that the number of beds are none too many to meet the demands of those patients who are able to pay a remunerative and inclusive rate for their treatment.

The Acland Nursing Home has succeeded in paying its way, and is now upon a self-supporting basis. Heretofore the profits have been devoted to the extension of the Sarah Acland Memorial, which provides district nurses for the poor of Oxford. This work has grown to a remarkable extent. Starting in 1882 with one district nurse it now has a staff of 60 nurses, a fact which testifies to the wisdom of the choice of an object to memorialise Sarah Acland, whose whole life won for her the affectionate regard and admiration of all from the highest to the lowest. There was one very pleasant fact brought out at Oxford on Saturday. The leading feature in Sarah Acland's life was an entire abnegation of self as a guiding principle to be followed by all Christian workers. Character, as we have often pointed out, is the first essential of a good nurse. It is gratifying to know that the spirit of the gracious lady who is memorialised by this Oxford Nursing Home seems to have entered into the workers attached to it, for her principles have secured for them complete happiness in their work to the comfort of everybody with whom they may be brought into contact.

### Abdominal Surgery.

By Harold Burrows, M.B., F.R.C.S., Assistant Surgeon to the Seamen's Hospital, Greenwich, and to the Bolingbroke Hospital, Wandsworth Common.

### AFTER-TREATMENT AND COMPLICATIONS.

(*Continued from p.* 5.)

### Peritonitis.

There are three varieties of post-operative peritonitis. In the first place there is the mild inflammatory reaction which follows immediately upon any injury or manipulation of the peritoneum. To distinguish it from the severer infective forms, this reaction has been termed peritonism. Its symptoms —abdominal pain, thirst, rise of temperature, vomiting—follow immediately upon the operation and are transient only, lasting as a rule not beyond thirty-six hours.

The second kind of post-operative peritonitis is that which is merely a continuation of a pre-existing infection of the abdomen. For example, a case of perforated appendix with diffuse peritonitis has been operated on and yet continues to show unmistakable evidence of septic peritonitis.

Lastly, there are the cases in which the peritoneum has become infected in the course of the operation. In these the symptoms become marked at the end of the second or on the third day after the operation. That is to say there is an interval after the operation during which the patient's condition does not cause any particular alarm. It is important that this form of post-operative peritonitis be recognised at the earliest moment possible, for unless active measures be taken forthwith a fatal issue is to be expected.

### Intestinal Obstruction.

Intestinal obstruction is a complication which may ensue immediately after a laparotomy, or it may appear at any time subsequently, perhaps when many years have elapsed. If the obstruction is present within the first few days after the operation, it may be a matter of great difficulty to differentiate between the symptoms due to obstruction and those due to peritonitis and tympanites. Persistent vomiting with absolute constipation, especially in the absence of marked abdominal tenderness, is very suggestive of obstruction. With peritonitis, as well as with tympanites, it is rare to get absolute constipation, for a turpentine enema will nearly always lead to the passage of flatus.

A sequel of abdominal diseases and operations, which is not uncommon, is partial intestinal obstruction. This may be due to pressure on the bowel by a tumour. More often it is caused by kinking of a portion of the gut, or by neighbouring coils of intestine becoming adherent together so that peristaltic movements cannot be easily and efficiently performed. The symptoms are distension of the abdomen, colicky pains, and constipation. Treatment consists of massage of the abdomen, when this is possible, and great care to avoid constipation by means of careful dosing and dieting. Complete intestinal obstruction may occur at any time in these cases, and it is well to be aware of this fact, so that no precious time may be wasted if symptoms of this catastrophe subsequently arise.

### Hæmorrhage.

Internal hæmorrhage is a rare complication of abdominal operations. But whenever it does happen it ought, without fail, to be recognised or at least suspected. The symptoms are pallor, thirst, rising pulse-rate and respiration-rate, and above all, restlessness. The treatment will depend upon the nature of the bleeding. The surgeon who has done the operation will probably be able to form an opinion as to whether the bleeding is from a large vessel needing a ligature, or whether it is merely an oozing which can be treated without a second operation.

### Thrombosis. Pulmonary Embolism.

Thrombosis, or clotting of blood in a vein, sometimes follows an abdominal operation. The femoral vein is commonly affected, and as a result the affected leg becomes swelled. The treatment is to bandage the limb in cotton-wool and raise it upon an inclined plane, so as to assist the return of blood to the heart. The patient must be kept absolutely quiet and still at first, for there is a danger that a portion of the clot may become detached and be carried to the heart and from thence to the lungs. This complication is known as pulmonary embolism. The sudden lodgment of the clot in the lung causes severe dyspnœa, and, not infrequently, death from suffocation.

In the course of time a clot in the femoral, or any other vein, will either become absorbed, or, more frequently, will become adherent to the vessel-wall, so that it is no longer likely to become detached and cause pulmonary embolism. Pulmonary embolism sometimes occurs when there is no obvious thrombosis, and this is especially the case after operations on the female pelvic organs.

### Parotitis.

Inflammation and swelling of the parotid gland is an occasional sequel of abdominal affections. There are two parotid glands, one on each side of the neck. Each gland is immediately beneath the ear and behind the jaw-bone. Its function is to secrete saliva which is carried to the mouth by a duct opening on the inner surface of the cheek close to the second upper molar tooth.

The causes of parotitis are arrest of the secretion of saliva and infection spreading up the parotid duct from the mouth. Therefore to prevent the complication the patient's mouth should be kept as clean as possible, and the secretion of saliva should be encouraged, when possible, by giving the patient something to suck or chew.

If parotitis is present, a large hot fomentation should be applied in order to ease the pain, which is rather severe. Whenever any signs of suppuration appear, the gland should be incised without delay, or disastrous secondary complications may arise.

The parotid gland is the part affected in mumps (epidemic parotitis), and the appearance and symptoms of post-operation parotitis are similar to those of mumps.

### Cystitis.

Inflammation of the bladder is usually the result of infection by catheter. It is more frequent in women than in men, because in the former the bladder is difficult to empty completely with the patient in the supine position, even if the catheter be used. And the retention of some of the urine in the bladder, together with the inevitable infection by the catheter, are two factors very favourable to the production of cystitis. Endeavours should be made, by the strictest cleanliness, to avoid introducing micro-organisms into the bladder, and, when possible, the catheterisation should be performed with the patient on her side, so that the bladder can be emptied completely.

So long as the cystitis lasts, a daily specimen of the urine first passed in the morning should be preserved, in order that the surgeon may observe the amount of pus and mucus present and may notice any other special abnormalities in it.

As a rule the cystitis is not severe and soon passes off under treatment if the patient is able to sit up.

### Lung Complications.

These are to be feared mostly in old, feeble, and bronchitic patients. Several factors take part in the causation of post-operative bronchitis and broncho-pneumonia. The anæsthetic is apt to cause excess of bronchial secretion, and the exposure of the body during operation has the same effect; moreover, the pain caused by each attempt to cough prevents the patient from ridding himself easily of the mucus collected in his lungs. The treatment in the main is prophylactic. There is one point of prime importance. It is to avoid all unnecessary exposure of the patient to colds and draughts during the operation, while he is being conveyed back to bed, and afterwards. In addition the patient should be propped up in bed at the earliest moment feasible after the operation. It is well, also, to alter his position frequently, allowing him to lie first on one side then on the other, to encourage him to cough, and to administer suitable drugs.

*(To be concluded.)*

### The Nurses' Clinic.

### TETANUS.

Lockjaw is fortunately a disease so seldom met with that it is possible to pass through a three years' training without seeing a single case, so that a few words on the nursing of it may be helpful. It rarely falls to the lot of a private nurse to attend two cases in succession, more especially that both should recover. Yet this was my good fortune. The onset in each case was gradual, the first symptom being the inability to eat, followed a few hours later by stiffness of the jaws, which, by the third day, were quite fixed, also by a look of intense fear and a drawn expression of the mouth, and general arching of the body, the head being bent back.

In each case anti-tetanic serum was injected hypodermically by the doctor twice daily from the starting of the disease till the symptoms subsided on the tenth day, this causing very great irritation over the whole body, which was relieved by the application of a weak solution of lead lotion, afterwards being dusted with boric and starch powder. The patient should be isolated if possible, and kept in a darkened room, and should lie on a firm mattress which will not move when patient turns, as each movement may bring on a spasm. All doors in the house should be kept closed to prevent any banging, and strips of carpet or brown paper should be laid on floor to deaden sound, and if a fire is required wrap up the coal in paper before bringing it into the room, and no visitor other than doctor and nurse should be allowed to enter. In the early stages a spatula, or small piece of wood padded with wool, should be handy to put between the teeth to prevent the tongue being bitten during the spasms. As most patients are quite conscious during this illness and generally guess its nature, the nurse should endeavour to inspire the patient with confidence, and never betray any fear that she may have by her looks, always appearing hopeful. Feeding, which is of vast importance, is usually done per rectum, and consists of the ordinary nutrient enemata. Brandy also being ordered by the doctor, an attempt should occasionally be made (between the spasms) to give some fluid by the mouth, so as to give the muscles a chance of relaxing. Bromide is also ordered by the doctor in large quantities, and morphia to induce sleep. Constipation is usually present, and it is safest to give an enema saponis regularly than trust to aperients, which may not be swallowed. As the disease is often caused by flesh wounds, it is important that these should be attended to minutely and carefully if left to the nurse to dress, and the slightest change in their appearance should at once be reported to the doctor. There is seldom any fever attached to this disease, but pulse and respiration should be carefully noted, as there is danger of suffocation and exhaustion, from which the patient often dies, and only constant attention to all details of nursing will sometimes help the patient to a good recovery.

### Incidents in a Nurse's Life.

### A NARROW ESCAPE.

The lights were low in the ward when No. 3 night nurse came on duty; it was her first experience of night work, and she felt a trifle nervous. A case had just been brought in from the theatre—a severe amputation—who was feebly expressing a wish for something unattainable when Nurse approached his bed. He was a man in the prime of life, and it seemed a thousand pities that he should henceforth be so handicapped in the battle of life. Alas! it was the old old story, injury through drink, as Nurse afterwards discovered. Luckily there was not another serious case in the ward, and Nurse prepared to give most of her time during the night to Number Fourteen. Towards midnight the house surgeon made his round, and, much to Nurse's satisfaction, inspected the outer dressings of "Fourteen's" stump, which was carefully propped up on a pillow in the regular orthodox fashion. "Well, Nurse, it is all right now, but if it begins to bleed he may die in a few minutes, so be

INCIDENTS IN A NURSE'S LIFE—*continued.*

on the look-out for hæmorrhage; I will leave you a turniquet, which you must apply promptly if need arises," and, with a commiserating glance at Nurse, the house surgeon turned to leave the ward. "Please send a nurse round during the night, as I cannot leave the patient, and I am alone at this end of the corridor," Nurse called after him appealingly, and, with a promise to do as she wished, the house surgeon finally departed. Slowly the hours of that long night crept along, and many, many times Nurse carefully inspected her patient to see that all was right. Every now and then the pink jaconet (with which the splint was covered) struck terror to her soul in the dim light, as it could easily have been mistaken for hæmorrhage. Towards morning the anxious weary nurse was once more looking carefully at the still spotless dressings of "Number Fourteen" when the candle she carried in her hand accidentally set alight to some inflammable material which had been placed beneath the patient, and in a moment the mass was in a blaze. No time for thought of danger to herself, only one idea flashed through nurse's mind, and that was that her patient must be saved at all costs. With great presence of mind she seized the burning mass with both hands, threw it blazing upon the floor, and at great personal risk stamped upon the flaming material till nothing but a few blackened ashes and a dark stain upon the floor were left to tell of how narrowly an awful tragedy had been averted. When the thrilling moment was over and Nurse could speak calmly, she turned to her patient, who, to her surprise, had been closely watching her proceedings and greatly admiring her pluck and promptness. It was all over so quickly that it was difficult to realise the great danger in which both Nurse and her patient had been during a few terrible moments. "Number Fourteen" was safe, and appeared none the worse for having witnessed Nurse's heroic attempt to save his life. "You were plucky, Nurse," he fervently exclaimed; "but if you had been ablaze I should have come to the rescue, leg or no leg." Meanwhile Nurse was quietly breathing a whole psalm of thanksgiving that such courage and self-sacrifice had not been put to the test. To the end of her life she will never forget that lonely vigil and her narrow escape from an appalling catastrophe.

### Links in the Chain.

As children we were frequently told "a chain cannot be stronger than its weakest link," a doctrine which, to youthful minds, was at once irritating and depressing, but which, unhappily, cannot be disproved. Many of the links of nursing service have been enormously strengthened of late years, but is the chain as a whole very much stronger than it used to be? If not, which are the weak links and how can they be re-welded? Organisation is the word of the day with regard to every great institution, and carried to an extreme it forges links that are unable to bear the strain which life is sure to put upon them, sooner or later. Large institutions, and even large, well-managed families, often remind me of the celebrated village school where absolute perfection in the repetition of the Catechism was attained by assigning one answer to each pupil, with due respect to his verbal memory. On one occasion the Catechism was being repeated at an inspection, and the system worked with perfect smoothness as far as the words, "Rehearse then the articles of thy belief." There was an uneasy silence, and then a pupil held up his hand, "Please, sir, the boy as believes ain't here to-day!"

In a first-class hospital each nurse is necessarily part of a complicated machine: does she ever realise that the day will soon come when she will have to be complete in herself? Does she ever picture herself alone with a patient, when the nurse who puts up extensions so beautifully, the nurse who makes such wonderful poultices, the nurse whose forte is moving helpless patients, the nurse who is a skilled cook, and the nurse who knows all about getting ready for operations, will have vanished like "the boy as believes"?

To strengthen this dangerously attenuated link nurses must be willing to take every duty in turn, instead of showing a strong preference for doing the things that they can do well, combined with a tacit determination not to do the things that they do indifferently. They must look beyond the present moment, and welcome every chance of becoming "good all-round nurses," although the immediate result be blame and discomfiture.

Even when nurses have learnt their work practically it too often happens that they have so little knowledge of the principles on which practice is founded, that they are entirely dependent upon the elaborate and expensive apparatuses with which they have always been surrounded. Place them in a bedroom in a country vicarage, or in a boys' boarding-school, or a "third floor back," and they seem unable to evolve order and comfort, or to carry out any but the simplest orders. "There is nothing in the house to do anything with," they declare plaintively to the doctor, and I have known that much worried person go back to his wife and borrow until even her trained patience was exhausted; while if the nurses had looked about them and used their wits, they would have found all that they really needed on the premises, or have shown the relatives how to procure the necessary articles at a very small expense.

Lack of sufficient allowance for differences of individual constitution in another weak point. Rules as to strength of antiseptics, or aperients, or as to the heat of baths, or methods of preparing the seat of operation, and so forth, are excellent as a guide, but excuse no nurse from the duty of close observation as to results.

Diet is another uncertain link. Matrons and sisters as a rule pay far too little attention to the matter, and the nurses depending on them for instruction not merely remain in ignorance of this vital subject, but unconsciously imbibe the prejudice that, except in a few extreme cases, when the doctor will tell them every detail, diet is not of serious moment. I fear I must acknowledge that there are few mothers of families, from superior working men's wives upwards, who do not know more about feeding the aged, the young, the ailing and the convalescent than the hospital nurse of three or four years' experience.

Another weak point in modern nursing is that the nurse's interest is often pseudo-scientific, and her attitude to the patient lacking in simple human kindness. Whatever he may be to a doctor, to a nurse the sufferer must not be a "case." It must be an ever-present fact to her mind that each sick person is a complete human being, with human needs, affections, hopes, aspirations and duties. There are nurses who seem unable to realise that "cases" are parents and children, brothers and sisters, husbands and wives. They are like a French lady who said to me during the blackest months of the South African war, "It can make so little difference to you; you have a paid army." I asked, "Because we pay our men a shilling a day instead of a sou, can they be produced without fathers and mothers? Have you any reason to suppose that they are without brothers and sisters? I have four relatives out there, my friend has six, and the friend you saw me with yesterday has already lost six." But she was unshakeable in her belief that a paid army costs no tears, leaves no empty seats round the hearth, no widows, no orphans! One probationer of the type complained of told a woman who was about to undergo a dangerous operation, and was the mother of six young children, that she "loved operations, and enjoyed the excitement of getting ready for them." Naturally enough the patient was bitterly wounded by the girl's lack of feeling, and if she had been of an excitable disposition I think it would have injured her even as a "case."

In connection with this trait there is a great lack of hopefulness. The scientific reading of the nurses—much of it most imperfectly assimilated—is very apt to turn to pessimism. It is doubtful whether a doctor should prophesy, especially evil; it is certain that a nurse should not, and up to the very last she should work in hope and encourage the relatives to do the same. The need of working in hope is especially great among the poor. For want of it, many and many a patient, as doctors well know, has been allowed to "slip through" gates that never fairly opened and might well have been closed.

The last point to which I will refer is the almost excessive attention given to complicated surgical work, combined with a comparative indifference to sufferings which—however acute—are of a common and ordinary kind, easily curable if favourable conditions were maintained. To the individual, suffering can never be common and ordinary, and nothing will persuade me that our duty is not first to those who can be readily cured and returned to a life of activity and usefulness. "An interesting case," I have always told my probationers when they have made inquiries about unusual diseases or operations, "is when a respectable bricklayer breaks his leg, and by careful nursing you can shorten his illness and enable him to return to work before his hardly-earned home is sold up for debt. Only by a strange perversion of mind could such cases as you have mentioned to me be interesting to a nurse, whatever they may be to a specialist in morbid pathology."

### With a Patient in an Italian Hospital.

BY AN ENGLISH NURSE.

This hospital, the Ospedale Giovanni Evangelista, whither I accompanied a patient from an English nursing home, is under the government of the "Fratelli di fatto bene," or "Brothers of well-doing." The staff consisted of four "Brothers" (one of whom is the dispenser), the doctor—a very clever man—a young man who helped the doctor and who was apparently still studying, because occasionally he went into the "city" for exams., one very old woman, "Maria," who looked after the women, and a girl who helped her, "Giovanna."

### Its Beautiful Situation.

The Ospedale is situated close to one of the gates of the city, and when May came it was very interesting to watch the hundreds of sheep headed by their shepherd, the dogs bringing up the rear, come out to the mountains at the back of the Ospedale, where they remained for quite three months. We had a lovely view of the valley, while directly opposite is a mountain on the slopes of which in July soldiers came to camp, and every morning during coffee the band of the regiment discoursed sweet music, then occasionally on a "festa" the mountain was illuminated with Bengal lights, making a most wierd scene.

My patient was operated upon the morning after our arrival. He was chloroformed before leaving his bedroom, at my request, a most unusual proceeding in Italy. These "brothers" had never even heard of a woman being present at an operation upon a man, but I heard subsequently that they thought it was not a bad idea. At any rate, afterwards they always asked me to go to any operations which were taking place, and if I happened to be busy or asleep they would postpone it until I was at liberty.

### The Theatre.

I noticed that the "theatre" was very up-to-date in some things, while in others it was extremely primitive. For instance, the room itself was very small, roughly speaking about 18 feet by 6 feet, while the "table" was "one of the latest" as was the instrument case, but the instruments themselves were assorted, some old and some new, and the operators were wonderfully casual. One day a woman was on the table and the hypodermic needle was applied. When the operator came to withdraw the needle part of it was nowhere to be seen; but no one worried. I spoke about it afterwards, and the doctor said: "Oh! it does not do to get excited, the same thing happened a short time ago, but the woman went out and is all right now, so don't bother"; and knowing that I could do no good I learned in time not to do so. The room which is called the dressing-room is a place where the patients, resident and visiting, have their dressings done, and is four times as large as the theatre; it contains a steriliser, hot and cold water, and a museum.

### The Work of the Brothers.

These "brothers" have very comfortable rooms, but they spend very little time in them, for they work fourteen hours a day, and if there is a bad case among the men they have to sit up with the patient, or if attention is only needed once or twice during the night they sleep on a bed next to the patient. If among the females there is a bad case one of the assistant women sits up, whilst one of the brothers occupies a bed in one of the men's wards, so as to be at hand if it is found necessary to call him. The latter wear cassocks and white over-alls, and look very clean and tidy, so different to the old dispenser, who took snuff, which left its mark down the front of his robe, sleeves, etc. I think he had never washed or shaved more than once a fortnight for some years. The head, if such he may be called, was a German. When the doctor and his assistant were out their "fratello" officiated, and he always came for me to help him, and many were the discussions we had as to whether this or that was a sprain, a dislocation, or a fracture until, much to my amusement, I earned for myself the name of "La Santa Angelica." I think the truth of the matter was that they liked to feel a woman's sympathy, a thing they had not heretofore been used to.

### A Strange "Home Sister."

The kitchen is a huge place with tables all round and stoves in the centre, and an assortment of copper utensils, and, for an Italian kitchen, everything was clean and tidy. The greatest drawback was the cook himself. He was an outsider, and sometimes went out and did not come back again for hours. Previous to his appointment the cooking had been done by a brother, but the doctors would not repeat that experience because he was always at his prayers, and therefore could not attend to his work. Another brother acted as "home sister"; he saw to the diets, etc., and if there were any complaints to make he was the man we went to, but this we seldom did, for they were all so kind to us. The patients chiefly came from the surrounding mountainous

WITH A PATIENT IN AN ITALIAN HOSPITAL—*continued.*

villages, very simple, plain folk, who lived mostly on macaroni, vegetables and fruit, consequently, after the operations they were up and off almost before an Englishman would have recovered from the anæsthetic.

Successful Operations.

I have seen the most delicate uterine operations performed, after which an English woman has to lie without moving for weeks, but these Italian "contadini," or peasantry, will live on "slops" for, say, three days, and in ten days they walk about, and go out altogether at the end of three weeks. I marvelled, but the doctor explained that they were of much coarser fibre, had no nerves, etc.; the only thing he said that did not do well there was any case of "lung trouble," though he did not know why. One morning a boy of seventeen was brought in shrieking at 7 A.M. He had been very much in love with a girl, and her people would not give their consent to an engagement, so he shot himself, but evidently did more damage than he intended, for the bullet was somewhere in his intestines or bowels. They operated at once; and never shall I forget that operation. It was a boiling July day; the small theatre was full to overflowing, and to this was added the pungent smell of the chloroform. Eventually it was found that the bullet had perforated the spleen, and this they removed; the operator said that the patient was quite as well without it, except that he would be more liable to take any infectious disease. The surgeon was particularly proud of this operation, as he said "it had only been performed sixteen times in the world's history, and he had done it twice." I never had any opportunity of finding whether this was true. At the end of three weeks his people came to take the patient home. They had hired rooms in the town, and he attended every day to have the wound dressed.

The Italian Surgeons.

It is a pleasure to see an Italian surgeon work; for lightning rapidity I never saw his equal. I told this one all about our English hospitals, and left him with his chief aim in life to run his hospital on the same lines, but he said, "You English women have no feeling." I tried to explain to him that we had quite as much as Italians, but had more control over them. Then another thing he could not understand was that ladies could live in the hospital. He mentioned one hospital he knew where they went every morning and home again at night, but live in, oh, dear no!

### Courtesy in the Ward.

BY A SISTER.

To many nurses who go through their training in our great hospitals the principal aim and object of their life seems to be to just get through their prescribed work and go off duty. It is difficult to imagine what influenced them in becoming nurses at all; for these are not true nurses, and the only way they could become so would be to be forced for a month to suffer as a patient in their own wards. A real nurse will do her work as well and as quickly, her ward will be as spick and span; but she will always find time to think of her patients' happiness.

It should be the aim of every nurse to make the period spent in a ward the happiest part of a patient's life, even though that patient be on a bed of sickness.

Nurses so often do not realise what a power for good they may be to those who are under their care. In the rush of work and in the effort to get everything done to time it is difficult to learn to know one's patients; but it makes a great difference to the patient whether a nurse looks upon him simply as an "interesting case" or as a suffering brother or sister.

Two probationers come into the ward, each takes up her side dusting and polishing. One goes about her work in a brisk, business way, and when she has finished how proud she is of her lockers, how they shine, how tidy are her beds! But ask her how No. 4 slept; if Jenkins is feeling less nervous about his operation; if Daddy is looking forward to the afternoon which will bring his old wife to see him. What does she know? "Oh, I have not time to talk to them now" is the reply.

The other nurse does her side just as well, just as quickly; but how bright her patients look! While working and polishing she finds time for a cheery "Good morning, Jones; won't the wife be glad to find you sitting up?" "Well, Walters, how glum you look! Cheer up, the operation will soon be over, and it won't be as bad as you think; and we shall all be hoping that it will be successful. There, I'll just move your cradle and you will lie more easily." "Now, daddy, I am sure you have slept better to-night; you look so well," and so on. Each patient on that side looks forward to the hour when their nurse comes on duty.

I once knew a nurse who, in a small but busy ward, always managed during some part of the day to read a chapter of some bright tale to her men, and on Sunday the good old Book was chosen.

I never knew how she found time, but I do know her influence for good was very great. The same nurse, when in the casuals, collected magazines to give to the patients to look at while waiting, thus by a little trouble changing the dreary hours into a bright spot of the day; and she was well repaid by the smile of greeting and "Thank you, nurse."

Again, in the matter of feeding, always find time and take the trouble to find out how your patient likes his food—whether he likes milk hot or cold, beef-tea salt or without, and humour these little fancies.

Some nurses seem to have great trouble in making their patients take their food. How monotonous they become, milk, beef-tea, beef-tea, milk! But even these may be looked forward to if brought with a genial smile, just ready to drink, not too hot, and flavoured just as one likes, placed in the hand in a comfortable position for drinking, not put down just out of reach. Always make a practice of tasting all food whether solid or liquid before taking to a patient, to be quite sure it is just right for him. It means more trouble, but is well worth it.

One evening it was just time for service in the ward, chaplain was just coming in, and I saw a pained look on one man's face. The nurse on duty noticed it too. "What is it?" "Oh, nothing important, nurse," said he. "You must wait till after service; I cannot stop now." Poor fellow, his cradle had slipped, and was resting on a toe. How much good could that service have done him in unnecessary pain all the time, when a minute would have put all right, and he could have sat up and entered into the singing happily?

If nursing be undertaken from the highest motives and with the determination to make it a work of love, such little trifling kindnesses become as much pleasure to the nurse as to the patient.

### Oxford Acland Home.

OPENING OF THE NEW WING.

On Saturday afternoon last a large and representative company assembled at the Acland Home, Oxford, the occasion being the opening of the new wing containing operating, sterilising, and anæsthetic rooms, with lift and open-air balconies. The Home was tastefully decorated with palms and flowers. The ceremony was performed by Professor Osler, and amongst those present were the Vice-Chancellor (the President of Magdalene), Sir George Dashwood, Sir Henry Burdett, Vice-Admiral Sir W. Acland, Miss Acland, the Provost of Worcester, the Rector of Exeter, the Master of Pembroke, the Principal of Brasenose, Lady Teignmouth, and many prominent members of the University and the city.

Professor Osler read the following letter:—

"Balmoral Castle, October 11, 1906.

"Dear Miss Acland,—The Queen has seen your letter and enclosures, and desires me to congratulate you most warmly on the great success of an undertaking which must be so dear to your heart, and which bears your great and good father's name. The reports of the two branches of the institution are also most satisfactory. With every good wish from her Majesty for the welfare of the Acland Home,

"Believe me, yours truly,

"Charlotte Knollys."

Having observed that it must be exceedingly gratifying to receive this kind of message from her Majesty, Professor Osler went on to say that while the public hospitals and public institutions provided for the welfare of the poor the rich and well-to-do, and those who were not eligible for admission to the public hospitals, had also to be considered. It was for these that such an institution as this was specially required. A home of this description, erected in memory of Mrs. Acland and Sir Henry Acland, who for so many years was the honoured Regius Professor of Medicine in that University, was particularly useful for acute surgical cases, enteric fever, and nervous diseases. But there were local conditions that made the home very necessary. There were some 3,000 undergraduates at Oxford, and it was very important that, in cases of serious illness, there should be an institution in which they could be cared for. Until that day he did not know the reason why the University had not its own sanatorium. It was not necessary, because this home supplied the place, and it was very gratifying to know that so many of the undergraduates, when need arose, came to the home and received the benefit of its service.

Mr. Montague Wootten then announced that a friend who wished to be anonymous had offered to defray the cost of a glass balcony and an outside spiral staircase, and the offer was gratefully accepted. A hearty vote of thanks to Professor Osler closed the proceedings. Tea and coffee were afterwards served in the board-room by the nurses.

### The New Home of the Maternity Nursing Association.

The object of the Maternity Nursing Association is to provide good nursing, by means of fully qualified nurses, to poor women in their own homes during their confinements. It is a provident institution, as the patients contribute according to their means—the lowest fees being 10s. for a midwife and 5s. for a monthly nurse. The staff consists of a matron (approved by the Central Midwives Board), two midwives, and two or three pupils, who are trained for the Central Midwives Board examination. The work is rapidly increasing. The Association has recently moved its headquarters from 5 Little James Street, Gray's Inn Road, to 63 Myddelton Square, where the committee have succeeded in securing a very convenient house. Last month an "at home" was held in order to give the friends and supporters of the Association an opportunity of inspecting the new home. It contains four floors and a basement, and seems to be very well adapted for its purpose. On the topmost floor are the bedrooms for the two midwives, nice pleasant rooms decidedly lofty. This is a characteristic of all the rooms in the house, and is especially valuable in the pupils' two rooms, which are divided into cubicles, three to each room. Each of these rooms also possesses two good windows. The home was unusually full at the time of the meeting, as two sets of pupils had overlapped. Below the pupils' rooms are the matron's pretty room and one which has been set apart for the paying guest the Association is hoping to add to its establishment. A nice bed-sitting-room is already fitted up in anticipation of her advent, the bed being one of those useful pieces of furniture that serve as a couch by day and a bed by night. A screen in a corner hides the washing-stand. On the ground floor are the two sitting-rooms, and the cosy-looking dining-room, with its warm red paper is in the basement. The bath-room contains a geyser, so that a hot bath is always available at any time of the day or year.

On October 31 there is to be an Old Pupils' gathering. This is usually a very popular function and well attended. The annual sale in aid of the funds of the Association will be held at Holy Trinity Mission Room, Little James Street, Gray's Inn Road, on December 4. Contributions of dairy produce, cakes, tea, or other articles, are earnestly requested for this sale.

### Everybody's Opinion.

**[Correspondence on all subjects is invited, but we cannot in any way be responsible for the opinions expressed by our correspondents. No communication can be entertained if the name and address of the correspondent are not given as a guarantee of good faith, but not necessarily for publication. All correspondents should write on one side of the paper only.]**

### THE NURSES' HOSTEL.

"A. P." writes: I have to hasten my long-delayed thanks and gratitude for your excellent article and remarks on the "Nurses' Hostel." Perhaps you have observed that nobody has brought the charge of exaggeration or "not proven" against the writers. We have indeed been a company of long-suffering mutes, seeing our work depart, and with it our money. Surely it is damaging proof when a whole body of women unanimously condemn a thing so heartily! They would never have done so had they not perceived the wrong of it. First and foremost we are nurses. As such the public and doctors have a right to approach us in, say, emergencies, and no time should be lost on the telephone. "Yes, my dear," said a friend to me. "You know Miss Wood is a good woman: she would never have treated Miss Hulme in such a disgraceful, unjust manner if there wasn't something in it." Yes, I knew that was how it would end, but to my mind instead of taking a post in the country, and "living it down," Miss Hulme should try for redress in His Majesty's Courts of Justice.

"E. S. D." writes from Yorkshire: I was astonished and very grieved to read of Miss Hulme's dismissal in The Hospital. When an inmate of the hostel a short time ago, I heard one morning as we were about to sit down to breakfast that he new lady superintendent had just arrived, and on her entrance to the room I formed a very favourable impression of her general appearance and the kindly expression of her face, and although I was only a few days with her in the hostel, I liked her, and was looking forward to seeing her on my return. Some of the hostel rules seemed to me to be absurdly strict, for many nurses stay there while visiting London as they would do at any other hotel, paying so much per week according to their bedrooms, and why "strict discipline" should be enforced in an hotel I cannot quite understand. Nurses have, as a rule, trying lives, many have no homes and always go to the hostel when out of a post, and they naturally like to feel it is a home, and not an institution. My sympathies are with Miss Hulme, and I shall be most grateful to any nurse who knows of her whereabouts and who intends writing to the paper, if she will kindly give her address.

A Nurse writes: In answer to "J. M. T.'s" letter in last week's issue, in which she claims that a large portion of nurses remained loyal to Miss Wood and her colleagues, and "had perfect confidence that, whatever course she thought it right to take, she had good and sufficient reason for," I should like to say that I think she is mistaken. Many who wished and tried to be loyal to Miss Wood could not help being amazed at her treatment of Miss Hulme. Again, she says, "The malcontents numbered a large proportion of newcomers." True, but also many who had had long experience of the Hostel, and knew Miss Wood well. Of course, if "J. M. T." has personally received much kindness from Miss Wood she naturally would uphold her; but I think that she cannot claim that she is on the side of the majority. She goes on to affirm that Miss Wood gave "as full and clear an explanation of the whole proceeding as anyone who had brains to understand could desire." I fear that I and the majority of nurses now in the Hostel, and those away in various places who have heard of this matter, must plead a sad lack of brains, as we cannot see that any explanation at all has been given of the unjustifiable treatment of Miss Hulme, especially during the last week of her stay at the Hostel. No one who had committed a crime could have been treated worse.

"Not a Shareholder" writes from Monmouthshire: The Nurses' Hostel, Francis Street, having gained much notoriety detrimental to its welfare in the recent issues of the nursing journals, I wish, as a visitor of some four or five years' standing, to give it a little fair play, and will speak strictly from my own experience. The grievance of the telephone was a real one. All letters, telegrams, etc., have been most promptly forwarded, once only has a letter been wrongly re-directed. I have often left boxes, etc., unlocked, and have never had a single thing stolen. I have no experience of vexatious rules. The cleanliness of the house is unquestionable. Food is of the first quality, nicely though plainly served, and plentiful. Having tried some, and observed many ways of living between cases, so far I have found none that can be done as economically and with the same degree of comfort as at the Nurses' Hostel. My experience has been gained almost entirely at the South Block, but I know sufficient of the North Block to testify to its cleanliness.

"One of Miss Wood's Admirers" writes: I have been a resident at the Hostel between my cases for nearly ten years, and I have great pleasure in testifying to the kindness and consideration I have always received from Miss Wood and Miss Paul, and I regret their resignations. I can never thank Miss Wood sufficiently for founding the Hostel, and I believe that all the old residents are loyal to her and would endorse what I have said. Although about 600 nurses make their home there only about 50 signed the petition, which shows that the majority appreciate the Hostel in spite of its supposed faults and failings. If the next Lady Superintendent manages only half as well as Miss Wood "she will do well."

### ALL SAINTS' SISTERS AND ST. GEORGE'S HOSPITAL, BOMBAY.

"Trained in Bombay" writes: With reference to a paragraph in "Notes from India" in a recent issue of your paper regarding St. George's Hospital, Bombay, I wish to say that if "the sisters of All Saints' are a dream of the past" they have left a very excellent foundation for the present lady superintendent's improvements. I feel compelled, in justice to my old training school and the sisters of All Saints', under whom I trained—testimonials I have received from medical men even in England refer to me as one who thoroughly understands the duties of a nurse—to tell my sister nurses this side of the water of the vast work these same gentlewomen did when they undertook the nursing of the "European General Hospital"—as it was then called—from Government more than twenty years ago. The hardships and positive discomforts they endured in their stuffy so-called quarters, and the difficulties they met with at every turn in those early days, are known to few but themselves. The order and discipline they instilled into their probationers in their duties and ward work, their kindness and forbearance with the unruly and troublesome spirits they often had to deal with, is well and most gratefully remembered by one who is now herself the matron of a rising hospital in London. Four of the nurses trained by the sisters of All Saints' are members of Queen Alexandra's Imperial Military Nursing Service; three others are holding good posts in England; another wears the order of St. John of Jerusalem for her noble work among the plague-stricken creatures in Bombay. These nurses, including myself, are purely English, daughters or sisters of officers in the army, civil servants, or other officials in Government service in India. That Eurasians are now not taken on the staff of the hospital is the result of time. The sisters had to use the material at hand when they fell short of better. I often wonder why matrons and nurses are in England so narrow and so loth to acknowledge good in any nurse trained abroad. It is only in wielding the broom, polishing brass, and scrubbing that we fall short of the English-trained nurse. We in our turn could jeer at many of our nurse friends here, who know little or nothing of nursing liver-abscess cases, malaria, Malta fever, relapsing fever, plague, and cholera. The Indian-trained nurse is quite at home in all medical cases, infectious and non-infectious that flesh is heir to in England.

### THE DIFFICULTIES OF THE PRACTISING MIDWIFE.

"One who loves Midwifery Cases" writes: I have read with interest the letters headed "An Indictment of the Midwives Act." May I venture to say through your paper that the scarcity of midwives is largely due to the small pay. I have been practising six years on the District, but unless I got a stated salary from a committee, I could not exist. I should be only too pleased to work on my own account but several difficulties stare me in the face. First, the pay; secondly, the doctors who do not like midwives—and there are very few who do; thirdly, the rules of the Central Midwives Board are very rigid. I for one dread a court, and in spite of the utmost care, there is always the fear of sudden death. Doctors, too, are often not get-at-able in time when in outlying districts. There are also some dirty patients who prefer the Gamp, though others appreciate the new midwife. I am truly thankful to the Midwives Club for help and advice in these cases. I would much prefer to work apart from a committee if I could obtain a living wage, so would many more. Even on the district as nurse, I will not accept less than £1 per week, with furnished room. By the time the laundry, food, and other expenses are paid, to say nothing of uniform, and bicycle, there is quite little enough, and one cannot save much for old age.

### TWINS FOUR TIMES OVER.

The District Nurse at Silsoe, Ampthill, Bedfordshire, writes: I wonder if it would be interesting to you to know that I have lately had born in my district twin babies, this making the mother's fourth lot of twins. Her first babies were twins, and there have been twins at the last two births, two babies being only seventeen months old when the last twins were born, so now the mother has four babies that cannot run. In all she has had thirteen children, and is now only thirty-three years of age. The mother and babies are all well. Her husband is a Bedfordshire labourer earning about 13s. a week. I think this must be welcome information to those who are concerned about the declining birth-rate, and probably it would puzzle most people how to provide for a family on such slender means, as there are now at home eight children to be maintained, not able to earn anything.

